# Node Vulnerability under Finite Perturbations in Complex Networks

**DOI:** 10.1371/journal.pone.0020236

**Published:** 2011-06-16

**Authors:** Ricardo Gutiérrez, Francisco del-Pozo, Stefano Boccaletti

**Affiliations:** 1 Centre for Biomedical Technology, Technical University of Madrid, Pozuelo de Alarcón, Madrid, Spain; 2 CNR-Istituto dei Sistemi Complessi, Florence, Italy; Tel Aviv University, Israel

## Abstract

A measure to quantify vulnerability under perturbations (attacks, failures, large fluctuations) in ensembles (networks) of coupled dynamical systems is proposed. Rather than addressing the issue of how the network properties change upon removal of elements of the graph (the strategy followed by most of the existing methods for studying the vulnerability of a network based on its topology), here a *dynamical* definition of vulnerability is introduced, referring to the robustness of a collective dynamical state to perturbing events occurring over a fixed topology. In particular, we study how the collective (synchronized) dynamics of a network of chaotic units is disrupted under the action of a finite size perturbation on one of its nodes. Illustrative examples are provided for three systems of identical chaotic oscillators coupled according to three distinct well-known network topologies. A quantitative comparison between the obtained vulnerability rankings and the classical connectivity/centrality rankings is made that yields conclusive results. Possible applications of the proposed strategy and conclusions are also discussed.

## Introduction

From coupled biological and chemical systems, to neural networks, to social interacting species, to the Internet and the World Wide Web, the behavior of many natural, social and technological systems can be conveniently modeled as the dynamics emerging from networks composed of a large number of highly interconnected units. Recent studies have revealed that such systems are characterized by peculiar topological properties (relatively short distance between any pair of nodes, high clustering, and fat tailed distributions in the node's connectivity), starting a movement of interest and research in the study of complex networks [1].

Given the high level of heterogeneity in the nodes' connectivity, a central issue in the analysis of such systems was, since the beginning, the assessment of the nodes' centrality, and of the network's security and stability. The main aim was to properly rank each one of the networking units in terms of the response of the whole system under attacks or disfunctions of any type that may affect that specific element. In particular, a very important concept that was used to assess stability and robustness of the global behavior (or performance) of networks under the action of external perturbations (as failures or malicious attacks) is that of vulnerability.

So far, many different approaches have been proposed to define a measure for network vulnerability, relating it to, for instance, decreased cohesion and network fragmentation under random failures and attacks [2]–[5] and variations of the network efficiency after topological damages or improvements [6]. A formalization of the concept in terms of vulnerability functions that meet certain basic mathematical properties consistent with the intuitive notion of the vulnerability of a graph (somehow related to regularity and to the number of alternative edges existing between nodes) has also been proposed [7]. These early efforts were essentially devoted to the study of how certain properties of a generic graph are affected by changes in the topology, such as the accidental (random) or intentional removal of elements of the network.

In this paper, we introduce an alternative approach to the definition of network vulnerability, that connects it to the way the network dynamics abandons a collective (synchronized) state under the action of a perturbation acting on one of the nodes of the graph. Thus, the graph topology is assumed to be constant over the time scales that are relevant for the propagation of the perturbation, and we deal with the vulnerability of a given collective state (the synchronous evolution of the network), making this approach substantially different from the studies previously referred to. The strategy, albeit close in spirit to the studies on the linear stability of synchronized states in chaotic systems, differs considerably from those studies in that it provides a ranking of the nodes in a network in terms of the vulnerability of the collective state under *finite size* perturbations applied on them. Relevant applications can be found, indeed, in technological or infrastructural networks, where a practical issue is often to design the better protection strategy for each one of the units to avoid the spreading over the system of an occasional breakdown or intentional large damage. Furthermore, we will show that the approach is actually suggesting the definition of a *finite time* ranking of the units, thus offering a way to distinguish the most vulnerable network's nodes in all cases in which the goal is to repair or restore the network dynamics over a finite time.

## Methods

In order to illustrate our method, we refer to three different networking systems, each one made of 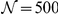 nodes and 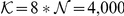 links. Topologically, the three networks are: 1) an Erdös-Rényi random graph (ER) [8], 2) a Barabási-Albert scale-free network (BASF) [9], and 3) a Configuration Model scale-free network (CMSF) [10]. Denoting by 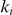 the degree of the 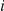-th node in the network (the number of its connections to the rest of the graph), we choose a CMSF with a degree distribution following the scaling law 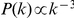, so as to make the network connectivity comparable to the case of BASF.

Without lack of generality, we consider each node 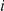 of the network to be represented by a vector state 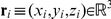, with internal evolution following the Rössler system [11] in one of its chaotic regimes, namely the one whose equations of motion are 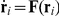 are: 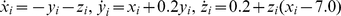.

Furthermore, we consider each node as diffusively coupled with its nearest neighbors in the graph, so that the network's evolution equations read as:
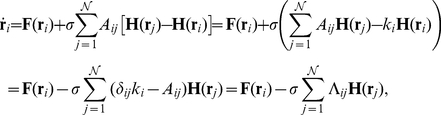
(1)where 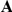 and 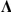 are, respectively, the adjacency and the Laplacian matrix of the network, and 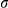 is the coupling strength. The adjacency matrix of an unweighted network is defined as the matrix with elements 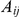 such that 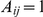 if there is a link incident in the nodes 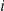 and 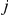 and 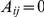 otherwise, which is a symmetric matrix with zero diagonal elements in the case of undirected simple graphs such as those considered here. The Laplacian matrix results from subtracting the adjacency matrix from a diagonal matrix with the node degrees along the diagonal. In equation (1), the connected nodes are linearly coupled through their 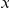 variable by the output function 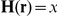. Notice that we consider all node systems to be identical. This, together with the zero row-sum condition associated with the Laplacian matrix, is warranting the existence of a synchronization state 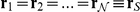, which is an invariant manifold.

As for the choice of the coupling constant 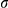, for each one of the considered networks we refer to the linear stability properties of the synchronization manifold 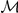. The Master Stability Function (MSF) approach [12] leads [after linearization and block-diagonalization of Eq. (1)] to 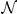 variational equations (one for each eigenvector of the Laplacian matrix) of the form 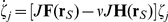, where 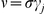 is the product of 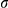 and the corresponding eigenvalue 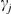 of 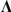, and 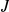 is the Jacobian operator.

Let us recall that in the present case all eigenvalues of 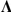 are real and non-negative. Furthermore, after ordering them by size (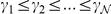), we have 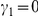 (corresponding to the synchronization manifold) and, as we deal with connected graphs, 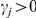 for 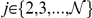, whose corresponding eigenvectors span (and form an orthogonal basis of) the space transverse to 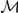.

For all three networks 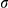 was chosen so as: *i)* 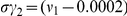; and *ii)* 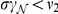 (where 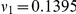 and 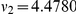 are, to our best numerical evidence, the first and second zero of the MSF, respectively). The result of this choice is that all the networks are considered in a dynamical regime in which the manifold 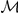 is slightly linearly unstable along the eigenmode corresponding to the second smallest eigenvalue of the Laplacian matrix (with an associated maximum Lyapunov exponent which amounts, according to our computations, to 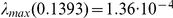), whereas it is linearly stable along all other eigenmodes. This allows for an unbiased comparison between the three considered topologies.

The MSF describes the local linear stability properties of the synchronized dynamics and, as so, it describes the evolution of infinitesimal perturbations affecting the dynamics on 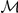. Since our aim is, instead, to study how the systems diverge from synchronization under the action of finite perturbations applied on individual nodes, and to relate this divergence to the topological features of the perturbed nodes, it is evident that a different strategy has to be followed.

To this purpose, the first step is to evolve a single Rössler oscillator from an arbitrary initial condition for a given time well beyond its initial transient. The final state of such an evolution is then taken as the initial condition 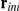 for the synchronous evolution of the full networked system (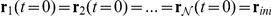).

An identical copy of the system is also started, with a finite perturbation applied on the arbitrary node 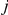. The initial conditions of this second system are 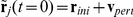, and 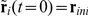 for 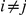. The chosen perturbation is of finite size, namely, it is a 4.0-norm vector (approximately 17% of the radius of the Rössler attractor) with the norm equally divided into the three components [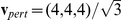 ]. It is the comparison of the networks states resulting from the simultaneous integration of both systems that allows one to monitor how the perturbed system's dynamics deviates in time from that of the unperturbed, synchronized system.

To quantify this deviation we use the global divergence rate (DR), a sort of Lyapunov exponent for finite perturbations and bounded time intervals. The DR for a perturbation applied on node 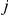 as a function of time is denoted as 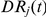. The DR is defined as the cumulative time average of the natural logarithm of the Euclidean distance (in 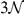-phase space 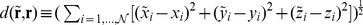) between the perturbed system state and the unperturbed (synchronized) system state divided by the norm of the initial perturbation.
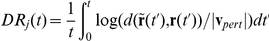



Interested as we are in the time evolution under perturbations of individual oscillators, we also consider the local divergence rate (dr). By 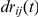 we denote the local divergence rate corresponding to the deviation from synchronization of the dynamics of the *specific node* 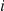 of the network for a perturbation applied on node 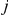. This latter quantity is calculated as the cumulative time average of the natural logarithm of the Euclidean distance (this time, in 3-space) between the specific 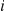-th node state in the perturbed and the unperturbed system, again divided by the norm of the initial perturbation.
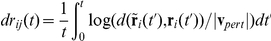



In the following, we report numerical results obtained with a classical 4th order Runge-Kutta integration algorithm, with double precision and 0.01 integration time step. Furthermore, all values of the local and global divergence rates shown in the Figures refer to a further ensemble average over 50 independent integrations of the system, each one corresponding to a different choice of the initial state 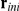, on top of which the perturbation is applied.

## Results

**Figure 1:**
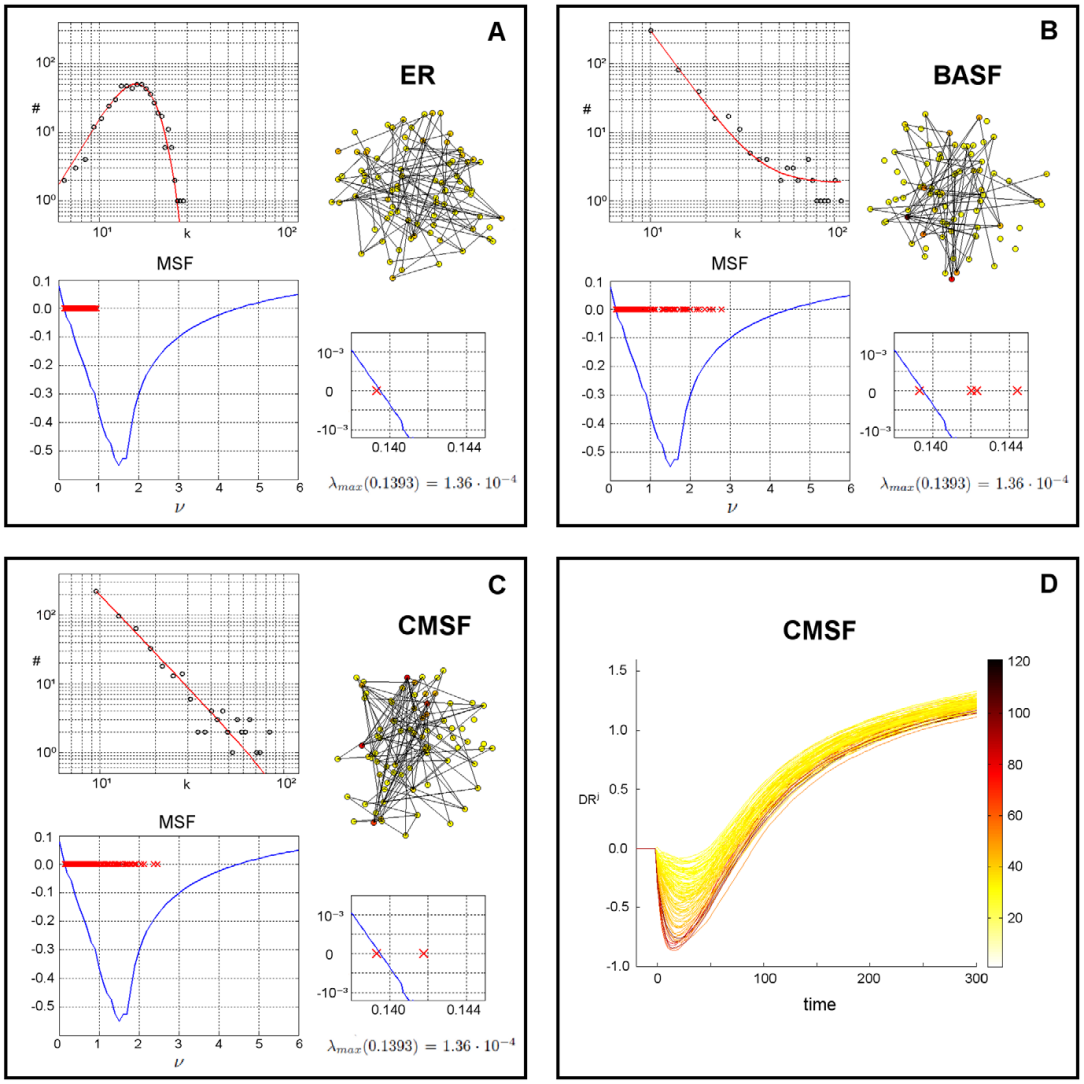
Topology and synchronizability (following the MSF approach) of the considered networks, and the effect of finite perturbations. A) ER random graph: degree sequence and least-squares fit curve (upper left plot), graphical representation of a subgraph containing 80 randomly chosen nodes (right plot) with color depending on node degree according to color bar in D), localization of the eigenvalues of the Laplacian matrix superimposed on MSF curve (lower left plot), and eigenvalues around the first zero of the MSF (lower right plot). B) Same for BASF network. C) Same for CMSF network. D) Divergence rates of the perturbed (global) dynamics from the synchronization manifold (see text for definition), for perturbations applied on 100 randomly selected nodes. Curves are colored according to the degree of the node upon which the perturbation is applied.

We start by showing in Figure 1 A, B, and C the main topological and synchronizability features of the chosen ER, BASF and CMSF networks. Precisely, the upper left hand side plot of each panel shows a histogram of the degree sequence, together with a least-squares fit curve (providing an estimate of the underlying degree distribution). The right hand side of each panel contains a sketch of a representative subgraph of the network, with nodes colored according to their degree following the color code of the bar in Figure 1 D. The lower left hand side, in its turn, shows the corresponding distribution of the eigenvalues of 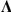 (red crosses) superimposed on the MSF curve (which is equal for the three cases in that it is independent of the topology). The spectra of the scale-free networks span a larger portion of the positive semi-axis than does the spectrum of the ER random graph, which turns out to be the easiest to synchronize of all three networks in spite of its homogeneity, a seemingly paradoxical fact that has been reported and previously explained in the literature [13], [14]. Another plot zooming on the area in the proximity of the first zero 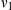 is shown on the lower right hand side, where one can see how the slightly linearly unstable regime is obtained by our choice of the coupling strength 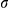.

Figure 1 D reports the DR as a function of time for the CMSF network. Each curve corresponds to 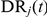 for a perturbation applied on node 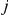, and the colors of the curves are indicative of the degree of the perturbed node 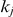 (to avoid cluttering, the Figure reports the evolution of DR for perturbations applied on only 100 randomly selected nodes). Initially, there is a sort of damping or dissipation of the perturbation up to reaching a minimum in the curve, followed by a steady increase that eventually approaches an asymptotic value. As shown in Figure S1, the same qualitative evolution of DR holds for different subsets of nodes spanning the entire degree range (Figure S1 C), as well as for the other two networks (Figure S1 A and B).

By a closer inspection of Figure 1 D (as well as of the analogous plots in Figure S1), it becomes clear that, in all cases, the DR's corresponding to perturbations applied on top of the most isolated nodes (those having the lowest 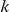) undergo the lightest damping and diverge rapidly from synchronization. Therefore, it is appropriate to refer to these nodes as *the most vulnerable* to a finite perturbation. On the other hand, as we inspect perturbations upon more and more connected nodes, the DR goes through a heavier damping and the divergence is slower. However, such a behavior happens to reverse at some point (at least for the BASF and CMSF networks, the ER having only nodes of low or intermediate connectivity), so that *the least vulnerable* nodes are not the hubs as one could have expected from the above observations.

While the data of Figure 1 D suggest a definition of a time dependent vulnerability, as the ranking of nodes (at a given time 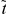) that follows the corresponding distribution of 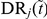, a remarkable result is that the curves appear to show, in fact, relatively few crossings between them. Precisely, the node ranking is almost conserved along the entire time epoch, a fact that can be used to simplify the operational relationship between vulnerability of a node under finite perturbations and its degree (or other centrality measures), by assuming the minimum of the 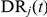 as a reliable measure of vulnerability. The idea is that the more negative the minimum is the larger the damping and the more inertia the system shows in escaping from synchronization.

**Figure 2:**
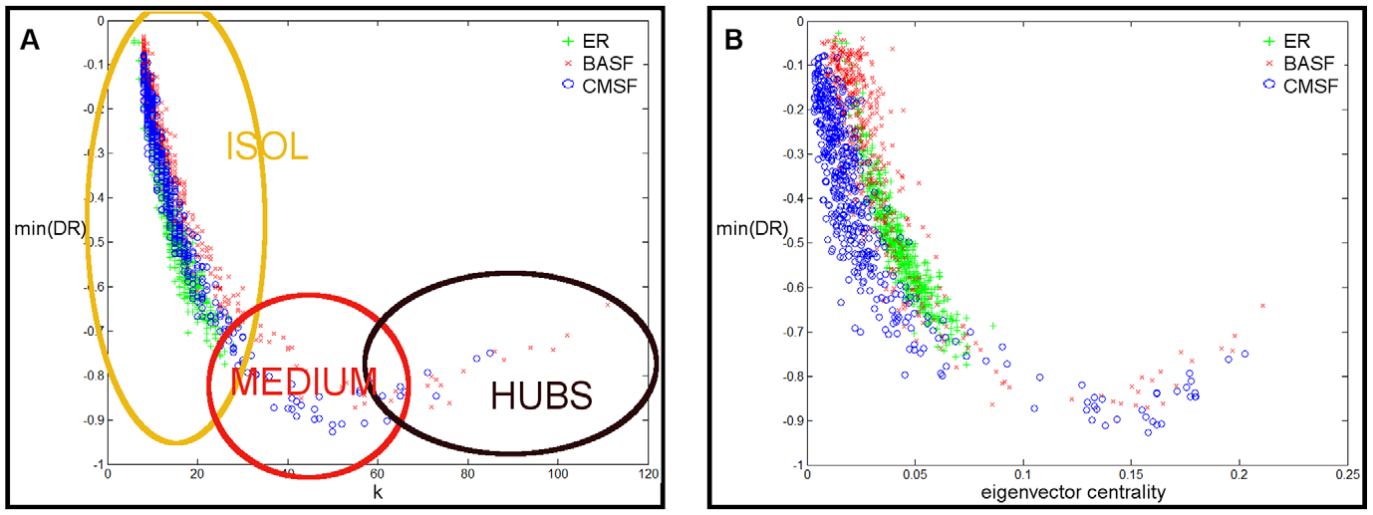
Minimum of the divergence rate as a function of connectivity/centrality. A) Minimum DR vs 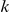. A simplifying partition of the nodes into three sets according to their connectivity (ISOL: isolated nodes; MEDIUM: nodes of intermediate connectivity; HUBS) is sketched for discussion of results. B) Minimum DR vs eigenvector centrality.

**Figure 3:**
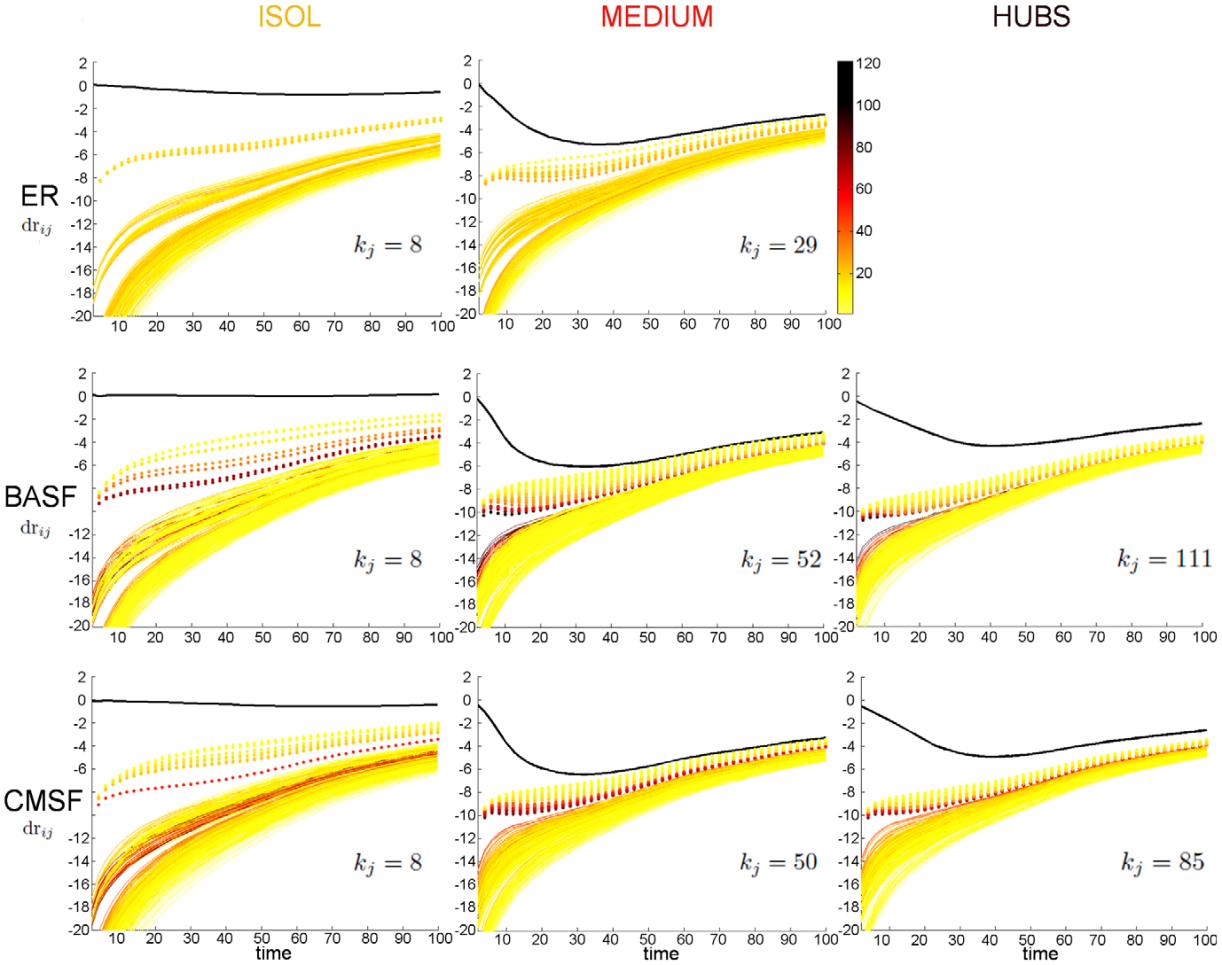
Propagation of the perturbation over the network. Local divergence rates 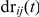 (see text for definition) vs time for the ER network (upper plots), the BASF network (middle plots) and the CMSF network (lower plots). The degree of the specific node 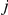 on which the perturbation is applied is reported on each plot. In each case, the divergence rate of the perturbed node, 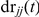, is shown in black, while the other (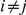) nodes' degrees follow the color bar in the second plot of the first row. Dotted lines correspond to the first neighbors of the perturbed node, continuous lines to the rest of the nodes.

Guided by the above discussion, we show in Figure 2 the minimum of each 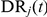 for all three networks as a function of 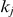 (Figure 2 A) and as a function of the eigenvector centrality [15]. Based on the qualitative time evolution of the global divergence rates and its dependence on the connectivity/centrality as seen in the Figure, we have grouped the nodes into three classes that roughly correspond to the isolated nodes (ISOL in the Figure), the nodes of intermediate connectivity (MEDIUM) and the hubs (HUBS). The transition between the isolated nodes and the nodes of intermediate connectivity has been further adjusted so as to correspond approximately to the point on the connectivity/centrality axis at which the stabilizing effect of the rest of the network becomes strong enough to cause a visible damping of the initial perturbation in the local divergence rate of the perturbed node 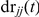 (such a phenomenon will be apparent in Figure 3).

As we anticipated, isolated nodes are the most vulnerable: a perturbation applied on them rapidly takes the system away from 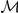 (see the region labeled as ISOL in Figure 2 A). As we perturb nodes that are more and more connected (those inside the region labeled MEDIUM in Figure 2 A) the escape from synchronization becomes slower: these nodes are less vulnerable to the perturbation, the speed at which the system desynchronizes is smaller, and, from the point of view of control of dynamical systems, a restoring or protecting action in technical applications could wait a bit more than in the previous case. In the case of the scale-free networks there are also nodes whose centrality/connectivity is still much higher (those inside the region labeled HUBS in Figure 2 A). As we perturb nodes of higher and higher degrees, we reach a point at which the trend is reversed into a situation where centrality and vulnerability to a perturbation are positively correlated quantities. This trend continues up to the most connected hubs as seen in the Figure.

The mechanism that underlies such a nontrivial dependence of the vulnerability on the centrality must be related to the way the perturbations are propagated over the network. The local divergence rates for individual nodes, 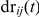 (where 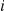 is the node under study and 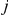 is the node initially perturbed) are then used to elucidate the situation. Figure 3 shows 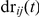 for every individual node 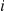, for perturbations applied on a few nodes which are either clearly isolated (left column), intermediately connected around the the region of lowest vulnerability shown in Figure 2 (middle column), or undisputed hubs of the network (right column); the degree of the perturbed node is shown on each plot. In each case, the thick black line corresponds to 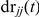 (the node under study is the node initially perturbed). The remaining curves corresponding to 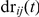 for 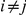 are represented as dotted lines whenever 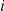 is a first neighbor of 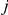 or as continuous lines whenever it is not.

Our results show that perturbations applied on very isolated nodes (those that are the most vulnerable) have some peculiar properties that become less distinct as we approach the boundary between isolation and intermediate connectivity. Indeed, the relatively light global damping (visible in Figures 1 D, S1 and 2) is seen to be due mainly to the fact that the perturbed node hardly suffers from any damping itself. As for the propagation of the perturbation, it is very heterogeneous, first affecting only the first neighbors in a way that seems to be inversely related to their degree (the most isolated are the fastest in abandoning the synchronized state), but eventually reaching the rest of the network. This second stage of the propagation occurs, instead, somewhat accordingly to the nodes' degree: hubs respond generally faster than intermediate or isolated nodes. As the region of intermediate connectivity is approached, the damping starts to be more and more prominent and the propagation of the perturbation more homogeneous.

When the perturbation is applied on nodes of intermediate to high connectivity, the generic behavior corresponds to a more homogeneous divergence of the full network from 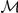. The way the individual nodes escape from synchronization as a function of connectivity is similar to that described in the previous paragraph. The reason why the hubs are more vulnerable than the nodes of intermediate connectivity, as previously observed, appears to be that for the hubs the damping turns out to be smaller, and the propagation over the network facilitated by a high connectivity makes the divergence from synchronization not only more coherent but also faster.

A complementary way to look at the propagation of the perturbations over the network is provided by the Videos S1, S2, and S3. In them, one can see how the natural logarithm of the Euclidean distance of each node in the perturbed BASF system to its counterpart in the unperturbed BASF system evolves in time when the perturbation is applied on an isolated node (Video S1), a node of intermediate connectivity (Video S2), or a hub (Video S3). As in all the Figures, averages across 50 independent integrations are used here. The same qualitative features are seen for perturbations applied on other nodes of similar connectivities in the BASF network, and also in the other two networks under study.

These results are robust for nodes of similar degree regardless of the specific topology of the considered network, suggesting that (at least for the networks under study) the differences in vulnerability under finite perturbations are largely (but not trivially) dependent on local (first-neighbors) properties. Moreover, the same qualitative features were found for other realizations of the three topologies of size 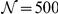 and 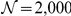, and also, in the CMSF case, for networks with a degree distribution 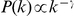 with 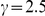 and 3.5.

## Discussion

In this paper we propose a novel method to study the vulnerability under perturbations (attacks, failures, large fluctuations) in large ensembles (networks) of coupled dynamical systems. Our method differs significantly from those of the classical studies on vulnerability in networks in that we do not address the issue of how the network properties change upon removal of elements of the graph. Rather, we consider a *dynamical* definition of vulnerability, namely, the vulnerability of a collective dynamical state to perturbing events occurring over a fixed topology. Specifically, we study how the collective (synchronized) dynamics is disrupted depending on the topological properties of the node in the network on which a perturbation acts. Moreover, we put the method to work by measuring the node vulnerability of three systems of identical chaotic oscillators coupled according to three distinct well-known topologies. We find conclusive results regarding the relationship between vulnerability under perturbations and node connectivity/centrality that seem to be robust and generally valid for different topologies.

The method consists of monitoring simultaneously both the original system and a copy of it subjected to a relatively large perturbation (a large additive term in the initial conditions of one of the networking units), and measuring the divergence rate between both systems, both globally and at the node level (if one is to study the propagation of the perturbation over the network in some detail). In some technological, physical, chemical or biological experimental settings where the systems are simple enough and highly controlled, a similar strategy could be followed with the same system used successively in two separate experimental runs, one for each initial condition.

Our numerical results highlight that there is a clear (yet non-trivial) dependence of the vulnerability on the nodes' degree and centrality. This dependence turns out to be highly robust and largely dependent on local properties, different topologies yielding essentially equal qualitative features. We have studied separately the action of a perturbation on isolated nodes, nodes of intermediate connectivity/centrality, and hubs. According to our results, a perturbation is taking the system out of a synchronized state most rapidly when applied on the most isolated nodes, and becomes less destructive as the perturbed node approaches the region of intermediate connectivity/centrality, showing a negative correlation between vulnerability and connectivity/centrality. After a certain value of connectivity/centrality is reached (where the vulnerability is at its lowest point), vulnerability and connectivity/centrality start to correlate positively, and the hubs of the network turn out to be as vulnerable under perturbations as some of the relatively isolated nodes.

When inspected at the node level, the divergence rates show that the propagation of the perturbation from the initially perturbed node to the rest of the network is very different depending on the connectivity/centrality of the perturbed node and of the other nodes. Some of the most conspicuous features are: a tendency for the most isolated nodes to stay away from the synchronization manifold since right after the perturbation, while recruiting more and more neighbors in a heterogeneous manner starting by the most isolated ones; and a tendency for the rest of the nodes to undergo some kind of damping after being perturbed and then diverge from synchronization with the rest of the network in a more homogenous way. Also, generally speaking, the first neighbors of the perturbed node seem to abandon the synchronized state with rates inversely proportional to their degree, whereas the rest of the nodes seem to do so at rates roughly proportional to their connectivity/centrality.

**Figure 4:**
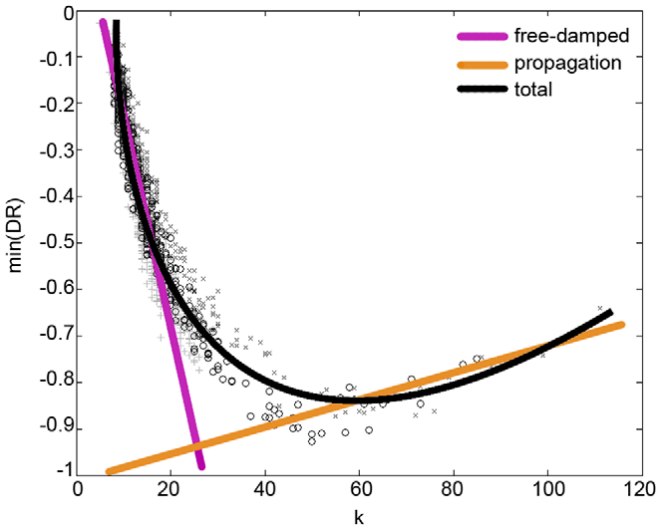
Illustration of a plausible interpretation of the results. The relationship between vulnerability and connectivity is assumed to result from the interplay between two opposing factors: 1) the more isolated a node is, the more free it is to remain perturbed, whereas the more connected the node, the heavier the damping it is subject to (magenta line); 2) the more isolated a node is, the weaker the propagation to other nodes, whereas the more connected the node, the better it is at propagating the perturbation throughout the network (orange line). The combined effect is represented by the black line, which is assumed to capture the main qualitative features of the numerical results.

These results can perhaps be interpreted as the interplay of two opposite forces or factors. On the one hand, there is the stabilizing influence of the other nodes in the network, which pulls the dynamics of the perturbed node onto the synchronization manifold and seems to be responsible for the damping of the perturbations. On the other hand, there is the fast propagation of information over such inter-connected networks, which helps the perturbation to reach all the nodes relatively rapidly (at least for the topologies considered, for which the geodesic distances are of necessity quite short). The first factor by itself would result in a monotonic decreasing dependence of the vulnerability on the connectivity/centrality; the second factor by itself would result in a monotonic increasing dependence of the vulnerability on the connectivity/centrality. The fact that we find a non-trivial dependence that is decreasing up to a minimum value and then increasing suggests that both factors (and probably others) are present to an extent, and become more or less prominent at different regions of the connectivity/centrality axis. Actually, results found at the local level (as shown in Figure 3) seem to agree pretty well with this explanation: isolated nodes are hardly subject to any damping but they propagate the perturbation relatively heterogeneously, nodes of intermediate connectivity are subject to a heavier damping and propagate the perturbation more homogeneously, whereas hubs behave qualitatively as nodes of intermediate connectivity, but with a still more efficient propagation over the whole network (which is assumed to make the damping lighter because the whole network is more rapidly taken away from synchronization). For the purpose of illustration, Figure 4 shows a simplifying, idealized version of this speculative explanation superimposed on the global results (shown in Figure 2 A). Further work along these lines is in progress to assess the full validity of the present interpretation as well as the generality of the results.

The relationship between the effect of large perturbations on a network of synchronized oscillators and the connectivity of the perturbed oscillator has been previously studied in the context of Kuramoto oscillators coupled following a scale-free topology [16]. Although the results are not strictly comparable with those reported here –in our paper there is an irreversible disruption of the (unstable) synchronized dynamics whereas Moreno and Pacheco work on a system where (asymptotically stable) synchronized states are spontaneously reached–, they find an interesting inverse proportionality between the vulnerability of the synchronized dynamics under perturbations (as measured by the resynchronization time) and the degree of the perturbed node. There is an obvious analogy between this finding and the damping of the divergence rates that we attribute to the stabilizing influence of the rest of the network on the node that has been perturbed, which, in our interpretation, should become more and more important as the perturbed nodes are more connected/central. The fact that the trend we see for small degrees/centralities in Figure 2 is reversed at some point is the effect of the propagation of the perturbation over the network, the second competing force. Nevertheless, the absence of any trace of an analogous effect in the results reported in [16] is not surprising, as only the first force is relevant in that context. Therefore these previous results are in good agreement with those reported here, and we believe they somehow lend support to our interpretation of the results in this study.

To summarize, the approach we introduce to the study of the vulnerability under finite perturbations in complex networks may be useful to unveil which nodes in a network are the most vulnerable to large damages or attacks, and thus those that are in more need of protection or rapid restoring action, when the collective dynamics is desirable, or those to be subject to an intentional attack if the build up of collective dynamics is to be prevented. For instance, the ranking of the nodes in terms of our measure of vulnerability could be of interest in the study of simulated networking systems and also experimentally in systems created for testing complex communication protocols (a perturbation could be a failure in one of the subsystems), neuronal cultures (an electric pulse applied on one neuron), etc. Moreover, we have tested the method numerically with three systems that are representative idealizations of many cases of interest. The results reported in this paper show a very definite dependence of the vulnerability on the connectivity/centrality of the perturbed node, which turns out to be relatively independent of the detailed coupling topology. This makes them potentially fit for extrapolation to a greater variety of systems. The protection of infrastructural networks, such as power grids, and the localization of the best spot for an intentional attack (electric current pulse or magnetic stimulation) meant to prevent or reduce undesired highly synchronized behavior in the central nervous system (e.g., Parkinson's disease, epilepsy, and other pathological rhythmic activities) are two relevant cases where the main results shown in our paper may be applied.

## Supporting Information

Figure S1 **Divergence rates of the perturbed (global) dynamics.** Divergence rates from the synchronization manifold (see text for definition), for perturbations applied on 100 randomly selected nodes. A) ER network, B) BASF network, C) CMSF network (subset of nodes different from that shown in Figure 0 D). Curves are colored according to the degree of the node upon which the perturbation is applied.
(TIF)

Video S1
**Propagation over the BASF network of a perturbation applied on an isolated node (** 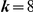 **).** The perturbed node is shown in the center surrounded first by a circle of first neighbors, further away by another circle of second neighbors, and so on, with lines between connected nodes. (In this and the other videos, to avoid cluttering, if there are more than 100 neighbors of a certain order, only 100 of them are randomly selected for visualization.) Azimuthal coordinate values (angles) are randomly assigned. Values along the z-axis represent the natural logarithm of the Euclidean distance between the state of the node in the perturbed system and its counterpart in the unperturbed system at the corresponding instant of time. Colors represent the degree of each node according to the scale shown in Figures 0 D and 0. Gray empty balls are just a visual aid to see the paths traced since the initial time.(MP4)

Video S2
**Propagation over the BASF network of a perturbation applied on a node of intermediate connectivity (** 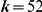 **).** See legend of Video S1 for a description of the objects in the video.(MP4)

Video S3
**Propagation over the BASF network of a perturbation applied on a hub (** 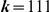 **).** See legend of Video S1 for a description of the objects in the video.(MP4)
