# Supplementary figures and images for: Node Vulnerability under Finite Perturbations in Complex Networks
Source: PLoS One. 2011 Jun 16;6(6):e20236. doi: 10.1371/journal.pone.0020236 (PMC3116827; doi:10.1371/journal.pone.0020236)

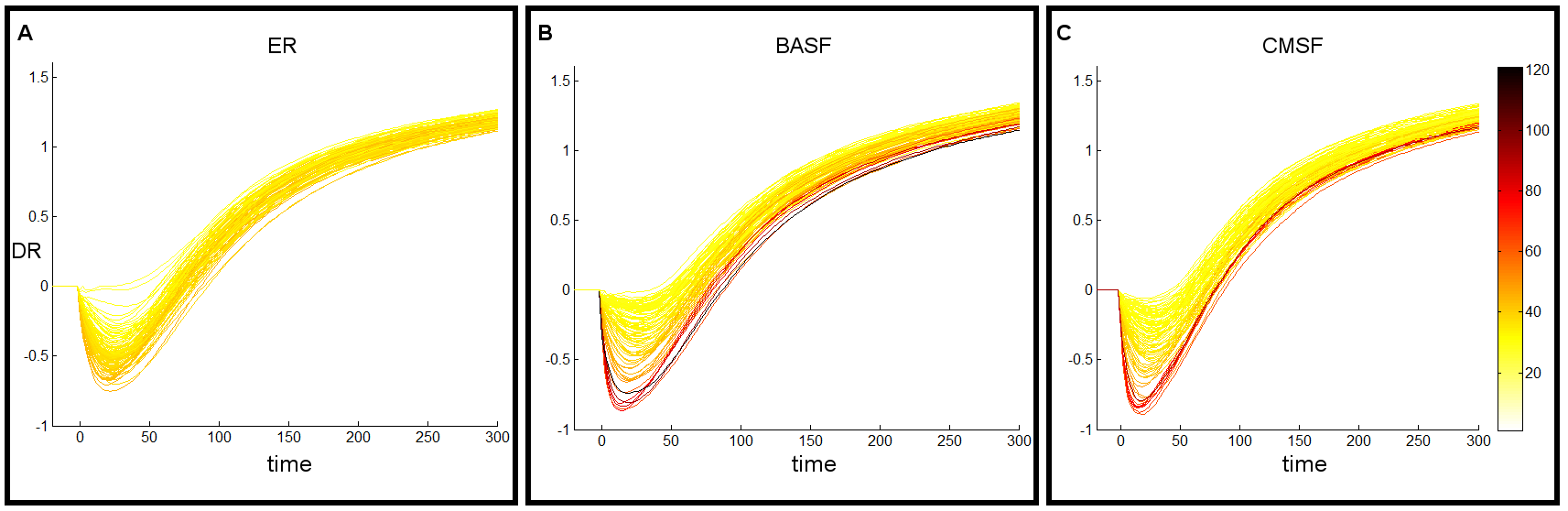

Supplement: Figure S1 — Divergence rates of the perturbed (global) dynamics. Divergence rates from the synchronization manifold (see text for definition), for perturbations applied on 100 randomly selected nodes. A) ER network, B) BASF network, C) CMSF network (subset of nodes different from that shown in Figure 0 D). Curves are colored according to the degree of the node upon which the perturbation is applied. (TIF) [file pone.0020236.s001.tif]
